# Coupling Analytical Models and Machine Learning Methods for Fast and Reliable Resolution of Effects in Multifrequency Eddy-Current Sensors

**DOI:** 10.3390/s21020618

**Published:** 2021-01-17

**Authors:** Sergey Kucheryavskiy, Alexander Egorov, Victor Polyakov

**Affiliations:** 1 Department of Chemistry and Bioscience, Aalborg University, Niels Bohrs vej 8, 6700 Esbjerg, Denmark; 2 Department of Physics, Altai State University, Lenina str. 61, Barnaul 656049, Russia; egav@bk.ru (A.E.); pvv@asu.ru (V.P.); 3 Institute of Strength Physics and Materials Science SB RAS, Tomsk 634055, Russia

**Keywords:** eddy current sensors, multifrequency eddy currents, machine learning, partial least squares regression, support vector machines, convolutional neural networks, deep learning, data driven approach

## Abstract

Eddy current (EC) measurements, widely used for diagnostics of conductive materials, are highly dependent on physical properties and geometry of a sample as well as on a design of an EC-sensor. For a sensor of a given design, the conductivity and thickness of a sample as well as the gap between the sample and the sensor (lift-off) are the most influencing parameters. Estimation of these parameters, based on signals acquired from the sensor, is quite complicated in case when all three parameters are unknown and may vary. In this paper, we propose a machine learning based approach for solving this problem. The approach makes it possible to avoid time and resource-consuming computations and does not require experimental data for training of the prediction models. The approach was tested using independent sets of measurements from both simulated and real experimental data.

## 1. Introduction

Non-destructive testing of metals and alloys usually involves estimation of their physical and mechanical properties, detection of cracks, corrosion and other flaws [1]. Quite often, the investigated material is hidden under a dielectric layer, for example anticorrosive coating or isolation material on a pipeline. In this case, the use of non-contact methods, such as eddy current measurements, is required.

Eddy-current testing (ECT) was proven to be an effective tool for the inspection of both magnetic and non-magnetic conductive materials [2,3,4]. It is mainly based on the variation of properties of the secondary magnetic field produced by eddy currents. The eddy currents are being induced within a conductive sample using an electromagnetic signal of a given frequency. The properties of the secondary field can be measured, for example, in form of impedance change of an EC-sensor (coil).

The impedance of the sensor depends on many factors, including conductivity, size and geometry of the sample, distance between the sensor and the sample, presence of various flaws, etc. To get more reliable results, the measurements are usually made for a set of activation frequencies, which gives a vector of the impedance values (EC-signal) [5]. Various research was devoted to establishing analytical or semi-analytical models, which make it possible to compute the impedance values depending on the frequency, parameters of the sample and the sensor, as well as the experimental conditions [6,7].

However, to use the eddy current testing on practice, a solution of the inverse problem is required [8]. In the present work we consider the following task—estimate a thickness of a sample and size of a dielectric layer on top of the sample, assuming that the exact conductivity of the sample is unknown but bounded by a relatively wide range. This task has no analytical solution, and solving the inverse problem is particularly difficult if the thickness of the sample is smaller than the corresponding skin-layer. So all three unknown parameters influence the impedance significantly.

One of the possible ways to overcome this problem is to use machine learning methods, where a mathematical model is trained based on data with a known response (the parameters of interest) and predictors (the measured EC-signals). After that, this model can be used for making predictions of the response values for a new set of measurements. This approach is often called “data-driven” or “soft modeling”, as in this case, the prediction models do not have any meaningful interpretation and, quite often, the prediction mechanism is hidden (e.g., in case of deep learning neural networks).

Once trained and properly validated, the machine learning models can predict the response values almost instantly, because the prediction, in most of the cases, requires just taking a cross-product of two or several matrices. The popularity and applicability of this approach are growing rapidly, especially in recent years. Thus in [9,10] authors successfully applied Partial Least Squares (PLS) and Support Vector Machine (SVM) regression for characterization of groove geometry (height and width) as well as orientation and size of cracks.

In [11] authors applied PLS regression for prediction of lift-off and conductivity of thick samples, where real experimental data were used both for training the regression model and validation of the predicted values. However, in this case models were made for thick samples, so the thickness was much larger than the corresponding skin-layer and therefore did not influence the shape of the EC-signals. This is a much simpler case than the one presented in this paper. To our knowledge, there is no research available where machine learning methods have been applied to resolve the effects of variation of the three parameters simultaneously.

The use of experimental data for training the machine learning models has its cons and pros. Thus if several parameters are unknown, this will require specially designed data, where the parameters vary at several levels each, which will result in hundreds of combinations. In case if non-linear methods, such as neural networks or gradient boosting, are considered, the amount of measurements should be even larger as otherwise, the models are prone to overfitting. Carrying out thousands of real EC-measurements is a very laborious and time consuming task.

In this work we propose to train the prediction models based on simulated data, generated using one of the analytical models, like it was also done in [9,10,12] and other research. However, the validation of the models is carried out using the designed experimental data to ensure that the models can be used for real life applications.

Narrowing down, the present work has the following objectives:

1. Assess the feasibility of using machine learning models for prediction of lift-off and thickness of conductive samples assuming that both are unknown. The conductivity of the samples is considered as unknown as well, but bounded by a relatively wide range of possible values.
2. Apply the models to real experimental data obtained independently using full factorial design to avoid confounding of the parameters of interest.

The manuscript describes the research and main results in detail. All calculations were made in R v. 4.0.3 [13] using packages mdatools [14], keras [15], e1071 [16], rootSolve [17] and CircularDDM [18].

## 2. Materials and Methods

To reach the objectives of this work, the following steps were carried out.

1. For a given set of activation frequencies, compute theoretical values reflecting changes in relative reactance and resistance of an eddy current sensor, located on top of a sample with given conductivity, σ, given thickness, *d*, and at a given distance (lift-off), *h*. Two subsets with simulated signals are created—one for calibration and one for validation of regression models.
2. Calibrate two regression models for the prediction of *d* and *h* correspondingly using the simulated data. Optimize the parameters of the models to ensure optimal performance on the validation set.
3. Acquire experimental data using a specifically designed eddy current sensor and a set of samples of different thicknesses at different lift-off values. The samples are made of two materials with different conductivity.
4. Apply the models to the experimental data for the prediction of *d* and *h* values. Compare the predicted and reference values.

This section describes the choice of methods for simulations and regression as well as the experimental setup in detail.

### 2.1. Experimental Setup

**Figure 1:**
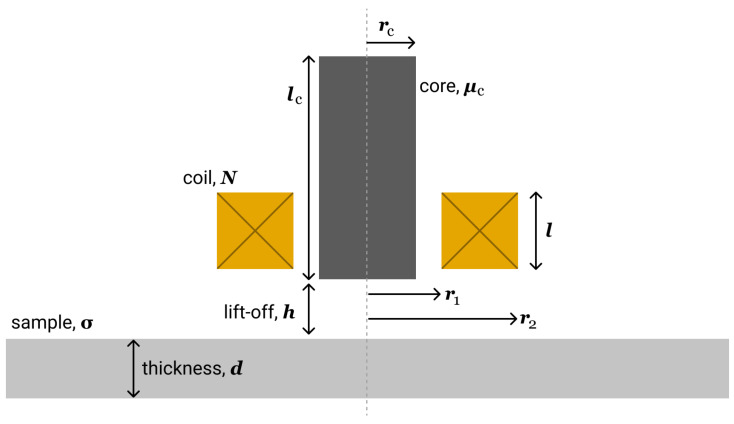
Sheme of eddy current (EC)-sensor and experimental set-up.

A schematic diagram of the eddy current sensor and the experimental setup, used in this work, is shown in Figure 1. A simple radial coil with ferrite core was used for both exciting the currents as well as for acquiring the EC-signals. Parameters of the sensor are shown in Table 1.

The measurement system included an AC power source, the sensor and a simple circuit coupled with the data acquisition unit. A computer’s sound card was used both for generating the AC signals as well as for reading the changes of voltage and current from the sensor using two channels of analog input of the sound card. The system itself and the measurements were controlled by a program created in the LabVIEW environment (National Instruments, Austin, TX, USA).

**Figure 2:**
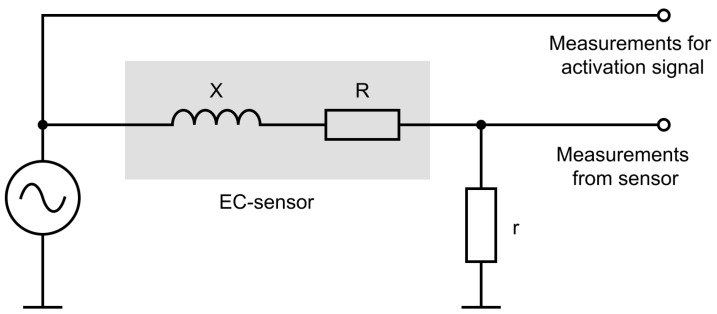
Shematic diagram of the measurement system.

A schematic diagram of the measurement system is shown in Figure 2 (adapted from [11]).The signal from the AC power source, used to activate the sensor, can be written in complex form as:

(1) $${\dot{U}}_{1}=U_{1}e^{j\phi_{1}}.$$

Here $U_{1}$ and $\phi_{1}$ are the amplitude and the initial phase of the signal correspondingly. The signal, measured from the sensor can be represented as:

(2) $${\dot{U}}_{2}=U_{2}e^{j\phi_{2}},$$

where $U_{2}$ and $\phi_{2}$ are the amplitude and the initial phase of the measured signal. After that, we can compute the resistance, *X*, and the reactance, *R*, of the sensor as:

(3) $$X=r\frac{U_{1}}{U_{2}}\sin\left(\phi_{1}-\phi_{2}\right)$$

(4) $$R=r\left[\frac{U_{1}}{U_{2}}\cos\left(\phi_{1}-\phi_{2}\right)-1\right].$$

Based on the measurements, an EC-signal can be represented in a form of changes of relative resistance, $\left(R-R_{0}\right)/X_{0}$, and relative reactance, $\left(X-X_{0}\right)/X_{0}$, of the sensor. Here $R_{0}$ and $X_{0}$ are resistance and reactance of the sensor without a sample, *R* and *X* are the values measured when the sensor was placed on top of the sample with a given lift-off. The measurements are carried out for a set of activation frequencies, resulting in a vector of values.

Two materials, duralumin alloy D16t (σ=15±0.5 MS/m) and aluminum-manganese alloy AMg5M (σ=22±1 MS/m) were chosen for the experiments. The materials were cut into several samples with thickness (*d*) varied between 1 and 8 mm. The width and the length of the samples were selected to be larger than $15r_{1}$, as required by the theoretical model, which is described below.

For each sample, the measurements were carried out at different lift-off values (*h*), varying from 0 to 2 mm. The lift-off was set by using dielectric films and measured with a micrometer. For every lift-off value, five measurements were taken at different positions within each sample and then averaged.

The reactance and resistance of the sensor were measured in a frequency range of 100–10,000 Hz with steps from 10 to 897 Hz (50 frequencies in total). The selection of the frequencies was based on the method described in [19], so the measured changes of voltage and current contained all needed harmonics, which reduces acquisition time significantly. Thus every EC-signal consisted of 100 values—50 for relative resistance and 50 for relative reactance.

### 2.2. Analytical Model and Simulation of ECT Signals

After preliminary consideration and comparison, the model proposed by Theodoulidis and co-authors [6,7] was chosen for simulating the eddy current signals. The model was shown to be efficient for a wide range of frequencies and the parameters of interests and can work with multilayer media, which is required for taking into account the thickness of the samples.

In its general form, the model allows to get an analytical solution for impedance of an axisymmetric eddy current ferrite-cored probe above a multi-layered conducting half-space. The solution is based on the truncated region eigenfunction expansion (TREE) method, which gives a possibility to replace integration by matrix operations [20]. Thus the impedance of a symmetric ferrite-cored probe over a multilayer conductor can be computed as:

(5) $$Z=j\omega \pi \mu_{0}\nu^{2}h^{T}Mh.$$

Here ν is a coil turns density, ω is angular activation frequency and $\mu_{0}$ is a vacuum permeability conctant.

The vector h is a column vector, whose values are computed using roots of specifically defined eigenfunctions and properties of the sensor (inner and outer radius of the coil, radius and magnetic permeability of the core).

The matrix M is obtained as a result of a cross product of several matrices, responsible for different effects. One of them is the reflection matrix, which in its turn, depends on the geometry and conductivity of the sample.

More details about the model and corresponding calculations can be found in [7]. The same expression can be used to compute the self impedance of the sensor, $Z_{0}$, by removing the effects of the conductor from the matrix M. The reactance, *X* and the resistance, *R*, can be then computed by taking the imaginary and the real parts of the impedance.

Since both h and M depend on roots of the eigenfunctions, their dimension, as well as the precision of results, depend on the number of the roots. In this work we use n=100 roots to achieve a proper convergence in the calculations, as proposed in [7]. This means that h and M have dimension of 100×1 and 100×100 correspondingly.

The implementation of the theoretical model was made in R. Function besselzero from the package CircularDDM was employed for finding first *k* positive zeros of the Bessel functions using Halley’s method. Package rootSolve [17] provided function uniroot.all, which was used to find roots for more complex functions within a given interval. The rest of the code was manually written based on linear algebra operators and functions BesselJ, besselY, integrate from the R distribution.

The theoretical model was used to generate signals for relative resistance and reactance for the defined set of activation frequencies based on characteristics of the sensor described in the previous subsection. Two sets of signals—one for training and one for validation—were created using combinations of the three parameters (thickness and conductivity of samples and the lift-off). A sequence of values (levels) was defined for each parameter as it is shown in Table 2. The values for the validation set spanned a bit smaller range and were shifted related to the values of the training set to improve the validation quality.

A full factorial design was used to create all possible combinations of the parameters resulted in 6615 signals for the training set and 5600 signals for the validation set. Although these numbers look overwhelming, there are several reasons justifying this choice:

1. The model to be created should work on a wide range of parameters. So a single model can be applied to experimental data obtained for samples with different conductivity, size and for different lift-off values, as we assume none of these characteristics is known a priory.
2. The use of full factorial design, in this case, is necessary to avoid confoundings—hidden cross-correlations among the three parameters. This in its turn is demanding for resolving the effects of conductivity, thickness and lift-off on the shape of the EC-signals.
3. A large number of measurements makes it possible to employ non-linear methods, which tend to overfitting otherwise.
4. The simulation is an inexpensive operation and requires (in this case) several hours of computational time. Once data is simulated it can be re-used for creating models based on different machine learning methods, optimization of the model parameters, etc. Acquiring experimental data of this size would require a much longer time and a lot of resources.

### 2.3. Regression Models

It was decided to employ three regression methods in this work.

Partial Least Squares regression (PLSR) is the simplest method, resulting in multiple linear regression model [21]. The main idea of PLS regression is to find a set of latent variables (PLS-components) oriented along specific directions in X- and Y-variable space [22]. If one projects the data points to these directions, the covariance between the coordinates of the projections in the X-space (X-scores) and coordinates of the projections in the Y-space (Y-scores) will be the largest possible.

Since the number of PLS-components is usually relatively small and the components are orthogonal to each other, this approach allows us to tackle two problems simultaneously—too many predictors (so-called curse of dimensionality) and the collinearity in predictors. This made PLS-regression de facto standard for solving regression problems using e.g., spectroscopic measurements. It was also used earlier for analysis of eddy current signals [10,11].

PLSR has several advantages over the other regression methods. First of all, it is fast, simple in implementation, and has only one parameter to optimize—number of PLS-components. The optimal number of components can be selected by using cross-validation or an independent validation set. Second, it provides a lot of exploratory tools, for example, to identify extreme samples or outliers, which do not share the same trend. PLS-regression also has tools (VIP scores, selectivity ratio and several others) for selection of the most important predictors [23].

It must be noted, however, that PLS regression implies a linear relationship between the predictors and the response variable. Therefore we can expect that it will perform poorly in the case of prediction of the sample thickness, which has a non-linear effect on the shape of the EC-signals. In order to tackle this issue, a use of non-linear methods must be also considered.

Support Vector Machines (SVM) is another popular supervised learning method that can be used for solving both regression and classification problems [24]. It was originally proposed as a method for discrimination of samples, which finds a hyperplane, separating points from two classes best possible, so the gap (or margin) between the classes, along the hyperplane, is maximized. In SVM, the orientation of the separation hyperplane is defined only by a subset of training measurements—support vectors, hence the name.

In the case of regression, the hyperplane is used as a regression model mapping the data points from X-space to *y*. There are two main approaches for the implementation of SVM regression, namely, ϵ-regression and ν-regression. The ν-regression, which is used in this work, has only one parameter to tune, ν, which is the proportion of the number of the support vectors to the total number of measurements in the training set. This parameter can be considered as an analog of the model complexity, so large ν may lead to an overfitted model.

Support Vector Machines is a linear method, however, a non-linear solution can be found by using a kernel trick, which maps the data points from the original variable space to a higher dimensional feature space using a kernel function [25]. Radial basis function (RBF) is one of the most used kernels for solving non-linear regression problems was employed in this work.

The third selected method is Convolutional Neural Network (CNN) regression [26]. It is a rather new method, based on the deep learning architecture [27]. CNN can be considered as an artificial neural network with several layers, where the first layer creates a convolution kernel that is convolved with the inputs over a single spatial dimension (in our case—frequencies).

One of the important reasons for the selection of the CNN regression method in this work was the ability to pre-train a CNN model. Pre-training is an initial step, where the CNN model parameters are optimized using a dataset, similar to the one the model is going to be used for, but more accessible. After that, the model can be fine-tuned using real data. The trick is that there is no need for a large number of real measurements for the fine-tuning step. This perfectly fits the purpose of the work—train a model using simulated data and then use it for predictions based on EC-signals measured experimentally.

The number and types of layers as well as their size and activation function give almost infinite possibilities for tuning. On the other hand, having too many layers leads to a huge number of parameters in the CNN model and, as a consequence, increases the risk of overfitting.

In order to make this risk smaller, we decided to use one of the predefined architectures and did not change either the number of layers nor their properties. This perhaps does not allow us to get the best solution possible, however in this case we did not have any parameters to optimize except the computation of gradient, which is done based on the provided validation set.

Thus, all three methods have only one parameter to optimize—number of components for PLS regression, number of support vectors for SVM regression and the way of computing gradient in CNN.

The quality of the models was evaluated graphically, using predicted vs. measured values plot, as well as statistically, by computing root mean squared error, RMSE:

(6) $$RMSE=\sqrt{\frac{\sum_{i=1}^{n}{\left(y_{i}-yp_{i}\right)}^{2}}{n}}.$$

Here $y_{i}$ and $yp_{i}$ are the reference (measured) and the predicted values for a given response variable (e.g., thickness or lift-off) corresponding to the *i*-th measurement.

It must be noted that the whole procedure, including optimization of the regression algorithm parameters for all three methods, took less than 5 min when running on a computer with 2.3 GHz 8-Core Intel Core i9 and 32 GB 2667 MHz DDR4 RAM under MacOS 11.0.1. The prediction is instantly fast (about 1 s for 5000+ EC-signals on the same computer).

## 3. Results

**Figure 3:**
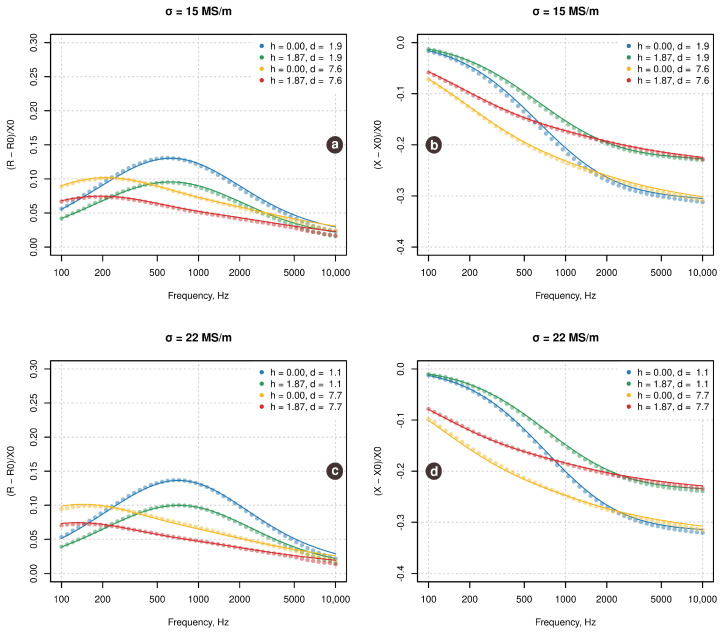
Relative resistance (**left**) and reactance (**right**) for the two alloys and selected combination of thickness and lift-off. Points show experimental data, measured using the EC-sensor. Curves show theoretical signals computed using model described in Section 2.2.

First of all, a qualitative assessment is carried out in order to see how well the theoretical model fits the experimental data. In order to do that, for each material, four measurements corresponding to the boundary values of lift-off and thickness were taken and theoretical EC- signals were computed using the analytical model described in Section 2.2. Figure 3 shows the experimental data (as points) and the computed theoretical signals (as curves) for each material.

Apparently, there is a quite good agreement between the experiment and the theory. However, one can observe a small but rather systematic deviation between the experimental points and the theoretical curves. This deviation can be explained both by uncertainty in the estimation of EC-sensor parameters (e.g., permeability of the core) as well as by imperfection of the theoretical model in general.

After exploratory analysis, three regression models were created for each parameter of interest (the sample thickness and the lift-off) using the methods described in the previous section. A simulated validation set was used for the optimization of the model parameters at this stage. The optimized models were applied then for the prediction of the experimental data.

R package mdatools [14] was used for PLS modeling. Package keras [15] provided R interface to Keras—a high-level neural networks application progamming interface supplemented with the Keras library. The support vector machine regression was utilized using implementation from the package e1071 [16].

**Figure 4:**
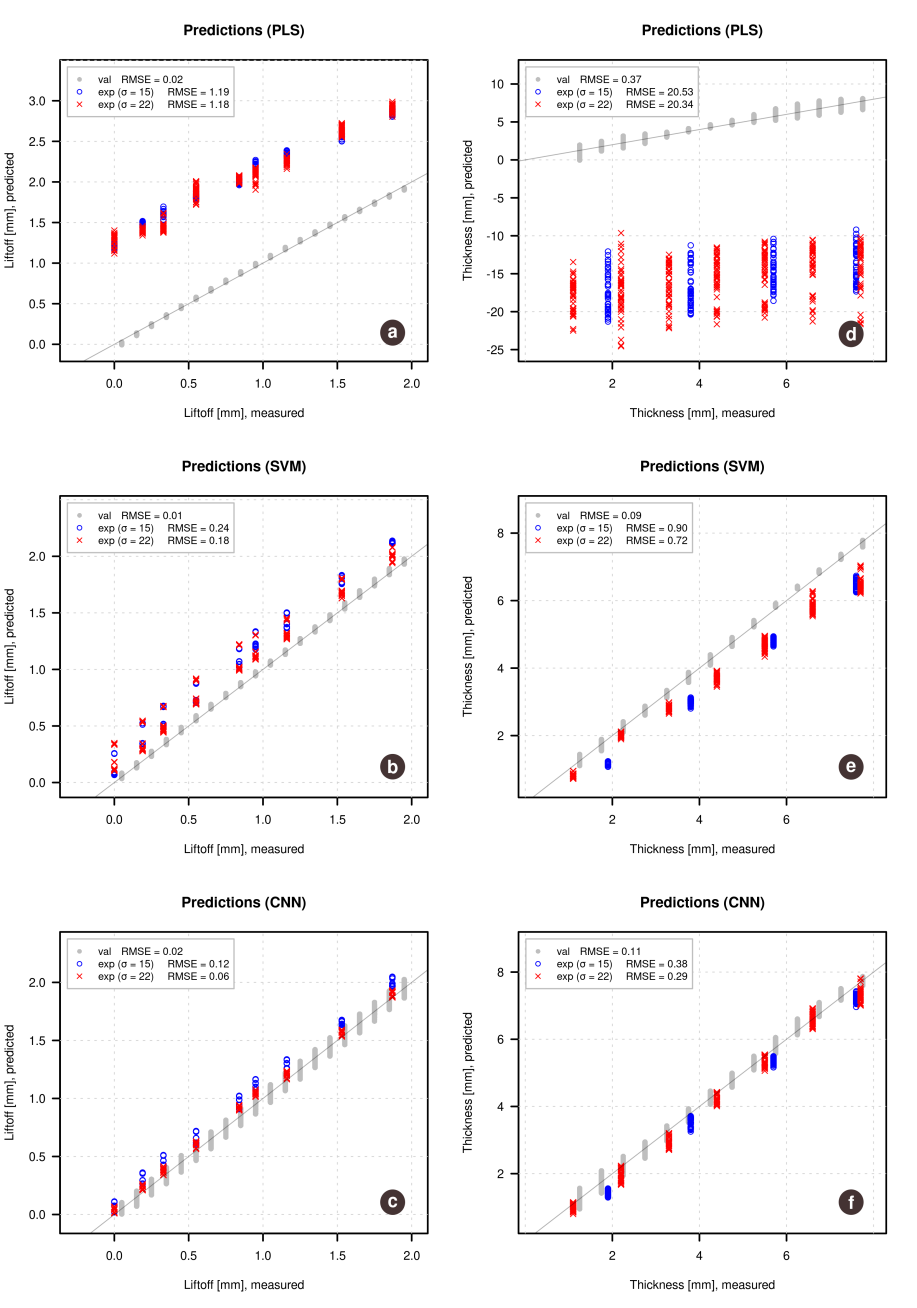
Predicted vs. measured plots for lift-off (**left**) and sample thickness (**right**) obtained using the three regression methods (a,d—PLS; b,e—SVM; c,f—CNN), optimized by the simulated validation set. Gray points show the results for the validation set, red and blue points show the predictions of the experimental data.

Figure 4 shows results in form of predicted vs. measured plots with corresponding RMSE values. The left plots demonstrate results for the prediction of lift-off while the right plots show results for the prediction of the thickness of the samples. Gray points correspond to the simulated validation set, on which the models were optimized. The blue and the red points correspond to the predictions made for the experimental data obtained for samples with conductivity σ=15 MS/m (shown as blue) and σ=22 MS/m (shown as red). Such representation allows us to see if the conductivity has any effect on the quality of predictions.

The figure clearly shows that Partial Least Squares regression, in this case, did not perform well either for the lift-off predictions nor for the prediction of the thickness of the samples from the experimental data. However, it can be also noticed that for the lift-off, the prediction error is mostly due to a bias, while the random error is relatively small. At the same time, the predicted values for the simulated validation set (gray points) are very close to the reference values.

The SVM regression performs much better, however, the predicted vs. measured plot also clearly shows the bias problem for predictions made on the experimental data—all predicted values tend to be by approximately 0.2 mm larger compared to the reference values.

The results obtained using CNN regression are the best in this case, although a small bias is also clearly visible. In case of lift-off one can also notice a small shift of the blue points (samples with smaller conductivity) related to the red points, which is reflected in the corresponding RMSE values (0.12 mm for samples with σ=15 MS/m vs. 0.06 mm for samples with σ=0.22 MS/m).

The prediction performance of the sample thickness is unacceptable in the case of PLS regression, demonstrating both bias and variance problems. The SVM regression model demonstrates decent predictions with a small systematic deviation—the thickness of the largest samples (around 8 mm) is clearly underpredicted, while the small samples (around 1–2 mm) have better prediction accuracy.

The regression model based on convolutional neural networks also demonstrates the best results in this case, although with a tiny bias. Overall, taking into account that the models were trained on the simulated data, CNN results demonstrating for prediction of experimental data it knows nothing about, can be considered as superior.

A deeper investigation of the models has shown that the main reason for the bias, especially in the case of PLS regression, is a small discrepancy between the theoretical and experimental results demonstrated earlier. Since the theoretical values, both for calibration and validation sets, do not have any noise or other disturbances, models being optimized based on the validation set trying to predict the theoretical values as good as possible (it can be seen by observing the predictions for the gray points) thus underlying the difference from the experimental data. In other words, using simulated data for optimization leads to overfitted models, especially in the case of PLS and SVM.

**Figure 5:**
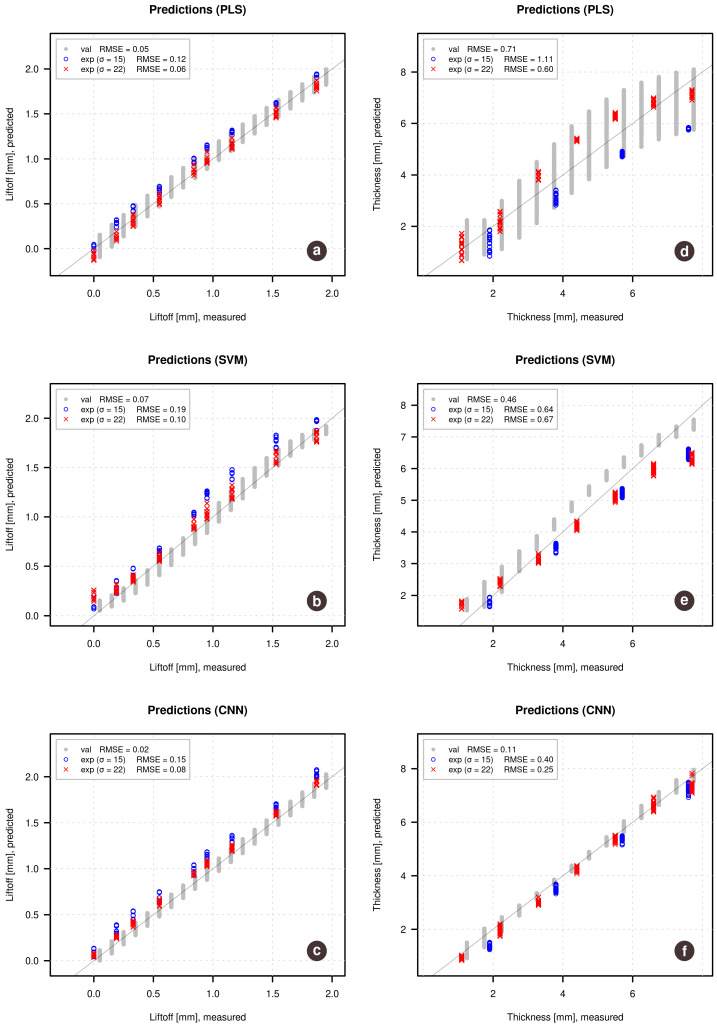
Predicted vs. measured plots for lift-off (**left**) and sample thickness (**right**) similar to what is shown in Figure 4 but for models optimized using the experimental data (a,d—PLS; b,e—SVM; c,f—CNN).

This issue can be tackled by employing the experimental data for the optimization of the model’s parameters. The models are still trained on the simulated data, but estimation of the optimal parameters (number of components, number of supporting vectors) as well as fine-tuning of the CNN regression models are made based on the experimental data. To check this assumption a new round of training/optimization steps has been carried out, the results are presented in Figure 5.

In case of prediction of the lift-off this was a game-changer for PLS regression model—it demonstrates the best prediction performance although with a tiny non-linear effect, which was also discussed in [11]. There is also a small shift of the blue and the red points observed earlier for the CNN model. The prediction bias disappeared but the variance became larger. This can also be observed for the gray points, the error of prediction for the simulated validation set and the experimental data are almost equal.

**Figure 6:**
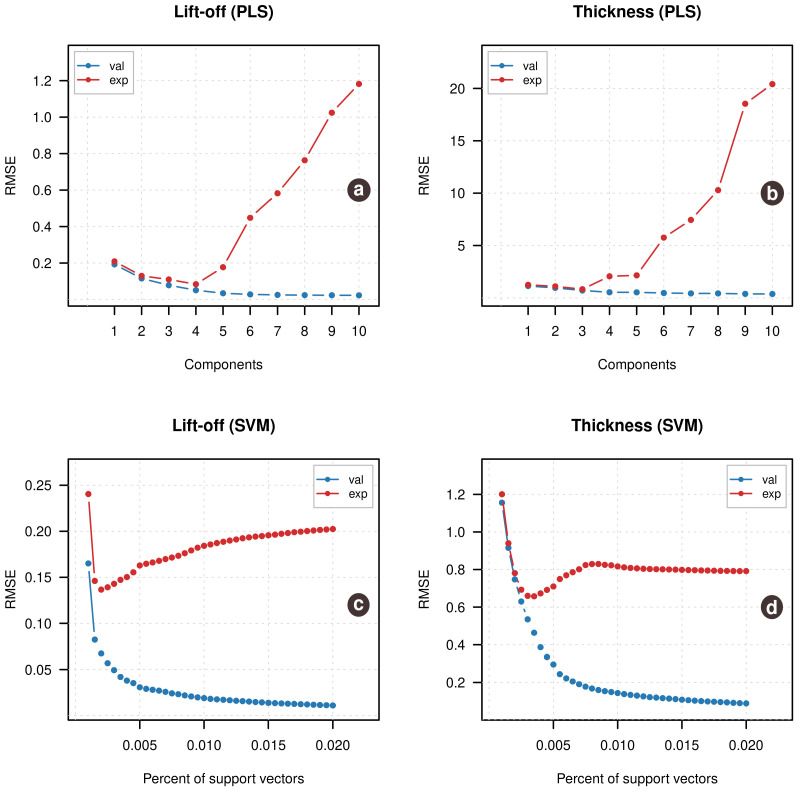
Root mean squared error (RMSE) vs. number of components plots for the Partial Least Squares (PLS) regression models ((**a**)—for prediction of lift-off, (**b**)—for prediction of thickness) and RMSE vs. ν-values for the Support Vector Machine (SVM) regression models ((**c**)—for prediction of lift-off, (**d**)—for prediction of thickness). The blue points show developing of RMSE for the simulated validation set, the red points show RMSE values for the experimental data.

The improvement is a result of changing the number of components in the PLS model from 10 (which was found to be optimal in the previous case) to 4. Two top plots in Figure 6 demonstrate how the root mean squared error values depend on the number of components in the PLS models both for prediction of measurements from the simulated validation set (shown as blue points) and the experimental measurements (shown as red points). In case of lift-off, there is a clear discrepancy between the two series starting from A = 4. Thus we can conclude that PLS regression strongly requires experimental data for optimization.

The SVM results also demonstrate a much smaller bias, however, in this case there is a clear sign of a small non-linearity on the predicted vs. measured plot. This is due to a much smaller number of support vectors used in the new model (0.2% of the total number of measurements in the calibration set vs. 2% found to be optimal in the previous case).

Two bottom plots in Figure 6 show how RMSE values depend on the selection of the ν parameter, similar to what is shown for the number of components in PLS-regression. Again, there is a clear minimum in both cases, when experimental data is used for optimization, while the simulated validation set did not allow to get the optimal value for the number of support vectors. Using the values obtained based on the experimental data improved the overall prediction performance for the SVM regression model (RMSEfor lift-off changes from 0.24/0.18 to 0.19/0.10).

The RMSE plots in the Figure 6 demonstrate a well-known problem of trade-off between underfitted and overfitted models. If a regression model is too simple (does not have a proper complexity), this leads to an underfitted model, so the fitting error is large. If model is too complex, this leads to an overfitted model, where the fitting error is very small, but model starts modeling noise in the calibration data and this gives a large sampling error when the model is being applied to a new set of measurements.

The overfitting can be captured only if a proper validation method is used. The best is to use a test set validation—based on new measurements with its own sampling error. However, simulated data in our case does not have proper sampling error, which, from our point of view, was the main reason why it did not provide a correct estimation of the model’s complexity. However, if the experimental data is employed for the validation, the overfitted models are spotted clearly, as shown on the plots,—in this case, the sampling error gets too large, which results in larger prediction error.

It is interesting, that the results for CNN remain almost unchanged in this case despite the fine-tuning. It must be also noted, that the results for the CNN regression may vary from run to run, because the model is trained using stochastic gradient descent. However, the variation is quite small.

The predictions of the thickness have also been improved significantly, however, PLS regression model can not tackle the non-linearity effect and failed to provide decent performance in this case. Support vector machines regression demonstrates a small improvement—RMSE values are decreased from 0.90/0.72 to 0.64/0.67. The model based on convolutional neural networks shows the best results with RMSE = 0.40/0.25, which is again almost identical to the results obtained without the use of the experimental data for optimization.

Table 3, Table 4 and Table 5 combine all information about the regression models, including tuned and fixed parameters as well as the prediction performance statistics.

In terms of relative errors, CNN has average relative error of 9% for prediction of thickness (min-max range 0.1–34%) and 19% for prediction of lift-off (range 2–65%). Average relative error for PLS is 18% (0.1–57%) for thickness and 14% (0.1–0.63%) for lift-off.

Most of ECT devices currently available on the market aim at detecting different flaws in materials rather than predicting their thickness and thickness of the dielectric coating. In case of just estimation of the coating thickness usually such methods as X-ray [28] or ultrasonic [29] are widely employed, however, devices utilizing the ECT as a main or supplementary method also exist, see for example [30] or [31]. The measurement error for the coating thickness provided by the producers varies between 1% and 3%.

## 4. Conclusions

The study shows great potential for use of a data-driven approach based on machine learning models to predict characteristics of conductive samples (thickness of the samples and thickness of a dielectric layer on top of them) based on multi-frequency ECT measurements in the case when all parameters of interest, as well as the conductivity of the samples, may vary and are not a priori known. The obtained results provide more evidence for a real benefit brought by using soft mathematical models for solving some applied physical problems when the exact analytical solution is not possible.

The presence of well established theoretical models for solving direct problems, like the one used in this paper, makes it possible to overcome one of the main drawbacks of the machine learning methods—collecting a arge amount of experimental data for model training. As it was also shown here, training a regression model on properly simulated data makes it possible to use it for the real experimental data with just a small drop in prediction quality. This also allows researchers to employ deep learning methods, such as convolutional neural networks, which require a large number of training measurements.

PLS regression is proven to be a good choice for the prediction of the lift-off (distance between a sample and an EC-sensor), which is easier to resolve from the other effects and which has an almost linear dependence on the shape of the measured EC-signals. However, it is necessary to use experimental data for optimization of the PLSR model, in particular, for selection of optimal number of components.

The use of more sophisticated methods, such as SVM regression and convolutional neural networks, makes it possible to solve non-linear problems, like the prediction of the sample thickness. One of the advantages of CNN regression, as shown in this work, is that when the CNN model is being trained and optimized using simulated data it performs very well even without the fine-tuning step. So strictly speaking, no experimental data are required.

In our opinion, the demonstrated results show the data drive approach a very good supplement to the state-of-art methods currently used in eddy current testing.

## Figures and Tables

**Table 1:** Parameters of the EC-sensor.

Coil		Core	
Inner radius, $r_{1}$	7.4 mm	Radius, $r_{c}$	5.1 mm
Outer radius, $r_{2}$	11.8 mm	Height, $l_{c}$	82.3 mm
Height, *l*	10.5 mm	Rel. permeability, $\mu_{c}$	200
Number of turns, *N*,	900		

**Table 2:** Parameters for simulation of the eddy-current testing (ECT) signals.

	Conductivity (MS)		Liftoff (mm)		Thickness (mm)	
	Cal	Val	Cal	Val	Cal	Val
Min	15	15.25	0	0.05	1	1.25
Max	25	24.75	2	1.95	8	7.75
Step	0.5	0.5	0.1	0.1	0.5	0.5
Number of values	21	20	21	20	15	14

**Table 3:** Parameters of Partial Least Squares regression (PLSR) models and corresponding RMSE values (Val model optimized using simulated validation set, Exp model optimized using the experimental data).

Parameter	Lift-off		Thickness	
	Val	Exp	Val	Exp
Number of components	10	4	10	3
RMSE (Val)	0.02	0.05	0.37	0.71
RMSE (Exp, 15)	1.19	0.12	20.5	1.11
RMSE (Exp, 22)	1.18	0.06	20.3	0.60
Algorithm	SIMPLS			
Preprocessing	mean centering			

**Table 4:** Parameters of SVM models and corresponding RMSE values (Val model optimized using simulated validation set, Exp model optimized using the experimental data).

Parameter	Lift-off		Thickness	
	Val	Exp	Val	Exp
Number of SVs, %	2	0.2	2	0.35
RMSE (Val)	0.01	0.07	0.09	0.46
RMSE (Exp, 15)	0.24	0.19	0.90	0.64
RMSE (Exp, 22)	0.18	0.10	0.72	0.67
Type	ν-regression			
Kernel	Radial basis function			

**Table 5:** Parameters of Convolutional Neural Network (CNN) regression models and corresponding RMSE values (Val model optimized using simulated validation set, Exp model optimized using the experimental data).

Parameter	Lift-off		Thickness	
	Val	Exp	Val	Exp
RMSE (Val)	0.02	0.02	0.11	0.11
RMSE (Exp, 15)	0.12	0.15	0.38	0.40
RMSE (Exp, 22)	0.06	0.08	0.29	0.24
Layer 1	1D convolution, kernel size = 2, 100×50, ReLU			
Layer 2	Flatten			
Layer 3	Dense, 50×50, ReLU			
Layer 4	Dense, 50×1, Linear			
Batch size	256			
Loss function	mse			
Epochs	100			
Optimization	Adam			

## Data Availability

The data presented in this study are available on request from the corresponding author.

## References

[1] Blitz J. (1997). Electrical and Magnetic Methods of Non-destructive Testing.

[2] Auld B.A., Moulder J.C. (1999). Review of advances in eddy current nondestructive evaluation. J. Nondestruct. Eval..

[3] García-Martín J., Gómez-Gil J., Vázquez-Sánchez E. (2011). Non-Destructive Techniques Based on Eddy Current Testing. Sensors.

[4] Ventre S., Calvano F., Pichenot G., Calmon P., Tamburrino A. (2011). ECT benchmark results for 3D defect signatures in industrial applications. NDT E Int..

[5] Egorov A., Polyakov V., Salita D., Kolubaev E., Psakhie S., Chernyavskii A., Vorobei I. (2015). Inspection of aluminum alloys by a multi-frequency eddy current method. Def. Technol..

[6] Theodoulidis T.P. (2003). Model of ferrite-cored probes for eddy current nondestructive evaluation. J. Appl. Phys..

[7] Lu Y., Bowler J.R., Theodoulidis T.P. (2012). An analytical model of a ferrite-cored inductor used as an eddy current probe. J. Appl. Phys..

[8] Albanese R., Rubinacci G., Tamburrino A., Villone F. (2000). Phenomenological approaches based on an integral formulation for forward and inverse problems in eddy current testing. Int. J. Appl. Electromagn. Mech..

[9] Ahmed S., Salucci M., Miorelli R., Anselmi N., Oliveri G., Calmon P., Reboud C., Massa A. (2017). Real time groove characterization combining partial least squares and {SVR} strategies: Application to eddy current testing. J. Phys.Conf. Ser..

[10] Ahmed S., Miorelli R., Calmon P., Anselmi N., Salucci M. (2018). Real time flaw detection and characterization in tube through partial least squares and SVR: Application to eddy current testing. AIP Conf. Proc..

[11] Egorov A., Kucheryavskiy S., Polyakov V. (2017). Resolution of effects in multi-frequency eddy current data for reliable diagnostics of conductive materials. Chemom. Intell. Lab. Syst..

[12] Le Bihan Y., Pávó J., Marchand C. (2008). Characterization of small cracks in eddy current testing. Eur. Phys. J. Appl. Phys..

[13] R Core Team (2013). R: A Language and Environment for Statistical Computing.

[14] Kucheryavskiy S. (2020). Mdatools—R package for chemometrics. Chemom. Intell. Lab. Syst..

[15] Allaire J., Chollet F. R Interface to Keras. https://keras.rstudio.com/.

[16] Meyer D., Dimitriadou E., Hornik K., Weingessel A., Leisch F. e1071: Misc Functions of the Department of Statistics, Probability Theory Group (Formerly: E1071), TU Wien. https://rdrr.io/rforge/e1071/.

[17] Soetaert K., Herman P.M. (2009). A Practical Guide to Ecological Modelling. Using R as a Simulation Platform.

[18] Lin Y.S., Heathcote A., Kvam P. CircularDDM: Circular Drift-Diffusion Model. https://cran.r-project.org/web/packages/CircularDDM/index.html.

[19] Egorov A.V., Polyakov V.V., Lependin A.A., Gracheva Y.I. (2017). Using signals of special form in multi-frequency eddy current testing. Optoelectron. Instrum. Data Process..

[20] Theodoulidis T.P., Bowler J.R. (2005). The Truncated Region Eigenfunction Expansion Method for the Solution of Boundary Value Problems in Eddy Current Nondestructive Evaluation. AIP Conf. Proc..

[21] Wold S., Sjöström M., Eriksson L. (2001). PLS-regression: A basic tool of chemometrics. Chemom. Intell. Lab. Syst..

[22] Haenlein M., Kaplan A.M. (2004). A Beginner’s Guide to Partial Least Squares Analysis. Underst. Stat..

[23] Andersen C.M., Bro R. (2010). Variable selection in regression-a tutorial. J. Chemom..

[24] Vapnik V. (2000). The Nature of Statistical Learning Theory.

[25] Schölkopf B., Smola A.J. (2002). Learning with Kernels. Support Vector Machines, Regularization, Optimization, and Beyond.

[26] Goodfellow I., Bengio Y., Courville A. (2016). Deep Learning (Adaptive Computation and Machine Learning Series).

[27] Schmidhuber J. (2015). Deep learning in neural networks: An overview. Neural Netw..

[28] Fischer Instrumentation Ltd. Coating Thickness Measurements. https://www.helmut-fischer.com/measurements/coating-thickness.

[29] Elcometer Ltd. Devices for Material Thickness Measurement. https://www.elcometer.com/en/ndt-equipment/material-thickness.html.

[30] DeFelsko Corporation PosiTector 6000. https://www.defelsko.com/positector-6000.

[31] Fischer Instrumentation Ltd. MMS Inspection DFT. https://www.helmut-fischer.com/products/mms-inspection-dft.

